# Endophthalmitis Caused by *Pseudomonas aeruginosa*: Clinical Characteristics, Outcomes, and Antibiotics Sensitivities

**DOI:** 10.1155/2022/1265556

**Published:** 2022-09-14

**Authors:** Jiaqi Lin, Shanshan Huang, Manli Liu, Lixia Lin, Jianjun Gu, Fang Duan

**Affiliations:** Zhongshan Ophthalmic Center, State Key Laboratory of Ophthalmology, Sun Yat-sen University, Guangdong Provincial Key Laboratory of Ophthalmology and Visual Science, Guangdong Provincial Clinical Research for Ocular Diseases, Guangzhou 510060, China

## Abstract

**Purpose:**

The aim of this study is to report the clinical characteristics, visual outcomes, and antibiotic susceptibilities of patients with *Pseudomonas aeruginosa* endophthalmitis.

**Methods:**

The medical records of patients with culture-proven *Pseudomonas aeruginosa* endophthalmitis treated from June 2013 to December 2019 were reviewed.

**Results:**

This study included 36 eyes of 36 patients. The clinical settings included ocular trauma (15/36), corneal ulcer (9/36), postoperative endophthalmitis (5/36), endogenous (3/36), and unknown (4/36). Sixteen patients underwent evisceration, 13 patients underwent pars plana vitrectomy (PPV), 2 patients were treated with only intravitreal antibiotics, and 5 patients did not undergo surgery. Only one patient achieved a visual acuity of 20/400, and the others had all counting fingers or below. The cultured *Pseudomonas aeruginosa* was 100% sensitive to gentamicin, tobramycin, amikacin, ciprofloxacin, and levofloxacin and, approximately 95% sensitive to meropenem, imipenem, and aztreonam.

**Conclusion:**

The visual outcomes of *Pseudomonas aeruginosa* endophthalmitis were very poor, and the evisceration rate remained high. *Pseudomonas aeruginosa* has good susceptibility to gentamicin, tobramycin, amikacin, ciprofloxacin, and levofloxacin.

## 1. Introduction

Endophthalmitis is one of the rarest and most destructive diseases due to its emergency, and it often results in irreversible visual loss [1, 2]. Endophthalmitis is classified as endogenous or exogenous according to the route of infection. Exogenous endophthalmitis is often caused by intraocular surgery, open globe injuries, intraocular foreign bodies, and corneal ulcers, etc [3]. Endogenous endophthalmitis is also called metastatic endophthalmitis because the pathogen spreads into the eye through blood and enters the eye through the blood-eye barrier and is reported to account for 2–41% of all endophthalmitis cases [4–6]. According to previous studies, endophthalmitis caused by gram-negative bacteria accounts for 10.7% to 29.1% of all endophthalmitis [7–12], and the visual prognosis is very poor [13]. Among endophthalmitis caused by gram-negative bacteria, *Pseudomonas aeruginosa* made up the largest proportion (23.0%–54.6%) [7–10, 14, 15].

*Pseudomonas aeruginosa* is a gram-negative bacillus commonly found in soil and moist environments. *Pseudomonas aeruginosa* endophthalmitis may complicate penetrating injuries of the eye, intraocular surgery, corneal ulcer, and spread from other sites of *Pseudomonas aeruginosa* infections. It is typically rapidly progressive and associated with severe vision-threatening and worse clinical outcomes [13, 16–18]. Thus, the purpose of this study is to review the characteristics of the clinical manifestations and vision acuity (VA) outcomes of culture-proven *Pseudomonas aeruginosa* endophthalmitis and the results of antibiotic susceptibilities.

## 2. Materials and Methods

### 2.1. Population

This retrospective study followed the principles of the Declaration of Helsinki and was conducted after being approved by the Ethics Committee of the Zhongshan Ophthalmic Center (ZOC) of Sun Yat-sen University. The requirement for patients' consent was waived given the retrospective nature of the study. Clinical records were reviewed for all patients with culture-proven endophthalmitis caused by *Pseudomonas aeruginosa* who were admitted to Zhongshan Ophthalmic Center from June 2013 to December 2019. The diagnosis of *Pseudomonas aeruginosa* endophthalmitis is correlated with clinical manifestations, including eye pain, decreased vision, vitreitis, and *Pseudomonas aeruginosa* culture, as proven in the laboratory. Medical history, demographic data, laboratory results, and treatment records, including surgical records, were collected and analyzed. Vitreous opacity was detected by an ocular B-mode ultrasonography scan. Evisceration operates in patients who are infected with severe suppurative endophthalmitis and who have no light perception and no possibility of vision recovery. Intravitreal injections of antibiotics were only used in patients with endophthalmitis or when there was a strong suspicion of infection, and vancomycin and ceftazidime were commonly used. Visual acuity was tested using an international standard visual chart. No light perception (NLP) is the visual acuity of an eviscerated eye.

### 2.2. Pathogen Isolation and Identification

The aqueous humor was aspirated from the anterior chamber through the limbus with a needle on a 1-mL syringe. Vitreous humor specimens were collected from the flat part of the ciliary body before antibiotic injection or vitrectomy through the pars plana. Corneal specimens were collected by scraping the base and edge of the corneal ulceration with a platinum spatula; one 2.5 ml syringe was used to extract the eye contents during evisceration surgery. The specimens were inoculated in bacterial culture medium such as blood agar or chocolate agar and prepared for Gram staining and were Giemsa staining. An automated system (VITEK 2 compact BioMérieux, Inc, Marcyl' Étoile, France) was used to identify bacterial isolates.

### 2.3. Antibiotic Susceptibility Test

In in vitro testing, the eye contents, corneal scraping material, corneal tissue, conjunctival sac secretion, or vitreous humor were cultured to confirm the positive culture results of *Pseudomonas aeruginosa*. The minimum inhibitory concentration method was applied to detect the sensitivity of *Pseudomonas aeruginosa* to *β*-lactam antibiotics, fluoroquinolones, aminoglycosides, macrolides and carbapenems. Antibiotic susceptibility was determined according to the method of clinical and laboratory standards research. The susceptibilities of the bacteria to these drugs were recorded as “resistant”, “intermediate” or “sensitive”. For the purpose of this study, being “intermediate” and being “sensitive” were both considered sensitive.

### 2.4. Statistical Analysis

The analysis of characteristics proportions and antibiotics susceptibilities were expressed as count and percentages. The age was summarized by median to present.

## 3. Results

A total of 36 patients (17 right eyes and 19 left eyes) were diagnosed with endophthalmitis and culture-proven *Pseudomonas aeruginosa*. The median age was 54 years old (range 6–85 years old). There were 26 men and 10 women. Among them, 2 patients had panophthalmitis, 4 patients had orbital cellulitis, and 13 patients had systemic diseases, including 5 cases of diabetes, 1 case of gastric ulcer, 1 case of kidney stones, 6 cases of hypertension, 1 case of kidney transplantation, and 1 case of hydronephrosis and syphilis. The clinical settings of patients with *Pseudomonas aeruginosa* endophthalmitis were trauma (*n* = 15), corneal ulcer (*n* = 9), postoperative endophthalmitis (*n* = 5, including 3 phacoemulsifications combined with intraocular lens implantation and 2 corneal transplantations), endogenous (*n* = 3), and unknown (*n* = 4). It cost patients 6.9 days on average during the course of hospitalizations. When patients were clinically diagnosed with endophthalmitis, intravenous antibiotics were used immediately. Except for one patient who was not suitable for intravenous antibiotics for kidney transplantation, only topical therapy and subconjunctival vancomycin and dexamethasone were administered. Ceftazidine or levofloxacin was most common, the proportions of patients who were treated by intravenous injections with ceftazidime, cefuroxime, levofloxacin and vancomycin are 29/36, 12/36, 19/36, and 2/36, respectively. Twenty-five patients received corticosteroid therapy, 19 received dexamethasone, 6 received prednisolone. Subconjunctival injections of tobramycin and dexamethasone were given to 7 patients. Ultrasound B-mode images showed turbidity of the vitreous in all patients, and 15 of them had retinal detachment. In the current study, 31 patients underwent surgical operations, 16 patients were eviscerated (only one patient previously received intravitreal antibiotics); 13 patients underwent PPV (including 5 patients had received intravitreal antibiotics), 2 patients received intravitreal antibiotics only. Among the 8 cases of intravitreal injection, 1 case was tobramycin, 1 case was ceftazidime, and 6 cases were vancomycin. There were 5 patients did not receive surgery: 1 patient was transferred to other hospital on account of suspicion of intracranial infection; infections were under control in 4 patients after treatments. Of them, two patients received corneal transplants. Among patients who underwent PPV surgery, 10 were filled with silicone oil in the vitreous cavity, 1 was filled with inert gas C3F8, and 2 were filled with balanced salt solation (BSS). Two patients had been treated with only intravitreal antibiotics without further surgery. The initial VA of all patients were hand motions (HM) or below. At last, only one patient had a visual acuity of 20/400, 3 patients had counting fingers (CF), 8 patients had HM, and 6 patients had light perception (LP), and 18 patients had NLP (including 16 patients with eviscerations). The detailed information of the patients is shown in Table 1 and the summary information are shown in Table 2.

Among 36 patients with culture-proven *Pseudomonas aeruginosa*, 3 patients were coinfected with *Pseudomonas aeruginosa* and other pathogens. In detail, one patient had mixed infections of *Pseudomonas aeruginosa* and gram-positive cocci, confirmed by smear examination. The second one was mix infections of *Pseudomonas aeruginosa* and candida. Another patient had mixed infections of *Pseudomonas aeruginosa*, amycolic acid, and corynebacterium sicca.

The susceptibility results of *Pseudomonas aeruginosa* are shown in Table 3. The cultured-proved *Pseudomonas aeruginosa* is 100% sensitive to gentamicin, tobramycin, amikacin, ciprofloxacin, and levofloxacin, with 96.7% sensitive to imipenem, 96.6% sensitive to meropenem, 95.2% sensitive to aztreonam, 75.9% sensitive to piperacillin-tazobactam, and cefepime, 75% sensitive to ofloxacin, and 74.3% sensitive to ceftazidime and carbapenems, and is 100% resistant to macrolide azithromycin medicine.

## 4. Discussion

A total of 36 patients (36 eyes) with culture-proven *Pseudomonas aeruginosa* endophthalmitis were reviewed. Of them, 41.7% had trauma, 25.0% had corneal ulceration, 13.9% had intraocular surgeries, and 8.3% had endogenous infections. The outcome of *Pseudomonas aeruginosa* endophthalmitis was still poor, and 16 patients underwent evisceration. Only one patient had a visual acuity of 20/400, and the other patients had a visual acuity of CF or below. The cultured *Pseudomonas aeruginosa* was 100% sensitive to gentamicin, tobramycin, amikacin, ciprofloxacin, and levofloxacin.

In our study, trauma was most common, followed by corneal ulcers and intraocular surgeries, which was different from previous studies reporting that *Pseudomonas aeruginosa* endophthalmitis is mostly caused by cataract surgery and corneal ulceration. For example, Eifrig et al. reported that cataract surgery accounted for 32.1%, corneal ulcers accounted for 25%, and trauma only accounted for 3.5% in the United States [13]. Similarly, Chen et al. reported that cataract surgery accounted for 15.3%, keratitis or scleritis accounted for 44.4%, and trauma accounted for only 6.9% in Taiwan [17]. Florida and Iran reported 33.3% and 85% of cataract surgeries, respectively, and there were no cases of corneal ulcers or trauma [18, 19]. Many factors may have contributed to this discrepancy because the etiology of endophthalmitis varies depending on the region and environment.

Endophthalmitis caused by *Pseudomonas aeruginosa* is a devastating intraocular infection and is always associated with poor visual outcomes. In the current study, the initial VA of all patients were HM or below. The final VA was 20/400 in only one patient; CF in 3 patients; HM in 8 patients; LP in 6 patients; and or NLP in 18 patients. Similarly, Falavarjani et al. reported that the final VA was HM or worse in 90% of patients, and evisceration was performed in 20% of patients [18]. Chen et al. reported that the final VA was LP or NLP in 86.1% of patients, and evisceration was performed in 50% of patients [17]. Sridhar et al. reported that the final VA was LP or NLP in 92% of patients, and evisceration was performed in 42% of patients [19]. All these studies indicated the visual outcomes of *Pseudomonas aeruginosa* endophthalmitis were generally poor, with a high rate of evisceration.

Currently, PPV has become the most common and useful surgical method for the treatment of endophthalmitis, and PPV combined with silicone oil can inhibit the progression of endophthalmitis [20–23]. In the current study, only 15 patients (36.1%) underwent PPV, which was much lower than that in the above studies. The low proportion of PPV might be related to corneal ulcerations, which could not undergo PPV. The previous studies showed that infectious ulcerations were associated with a high proportion of evisceration [24–27]. In recent years, endoscopy-assisted vitrectomy was considered an alternative treatment for endophthalmitis when patients are complicated by poor visibility through the anterior segment [28, 29]. As an ophthalmic endoscope had the potential to overcome the limitations of poor visualization and enhance the visualization of the posterior segment, which allowed the surgeon to perform vitrectomy safely and completely. Therefore, PPV is still considerable in the treatment of endophthalmitis, and silicone oil tamponades are also an important method to control endophthalmitis.

In our study, *Pseudomonas aeruginosa* was 100% sensitive to gentamicin, tobramycin, amikacin, ciprofloxacin, and levofloxacin, approximately 95% sensitive to meropenem, imipenem, and aztreonam, and approximately 75% sensitive to neomycin, piperacillin, cefepime, ceftazidime, and ofloxacin. Chen et al. reviewed 71 patients with endophthalmitis in Taiwan from 1997 to 2007 and reported that *Pseudomonas aeruginosa* was almost 100% sensitive to ceftazidime, cefepime, imipenem, and aztreonam, 94% sensitive to amikacin and 86% sensitive to gentamicin [17]. The susceptibilities of ceftazidime, cefepime, and imipenem were higher than our results, and the susceptibilities of amikacin and gentamicin were lower than ours. The most significant change is the decreased sensitivity of *Pseudomonas aeruginosa* to ceftazidime. Among our 36 patients, 29 patients intravenously used ceftazidime immediately after the diagnosis of endophthalmitis (before the culture results were released). The extensive use of ceftazidime might contribute to the increased resistance of *Pseudomonas aeruginosa* to ceftazidime. Falavarjani et al. reviewed 20 eyes of 19 patients with *Pseudomonas aeruginosa* from 2005 to 2015 and found 100% sensitivity to ciprofloxacin and imipenem [18], which is consistent with our results. However, they reported 88.3% amikacin, 83.6% tobramycin, and 76.5% gentamicin, which is lower than our results. The differences might be explained by the susceptibility to antibiotics changing with time.

The limitations of this study included its retrospective nature and relatively small size. Some of the initial origins of *Pseudomonas aeruginosa* endophthalmitis were not available. Furthermore, we only included cultured-positive cases, which could have underrepresented the overall etiological factors of *Pseudomonas aeruginosa* endophthalmitis. Nevertheless, our study provides valid data to describe the clinical characteristics, visual outcomes and antibiotics sensitivities of culture-proven *Pseudomonas aeruginosa* endophthalmitis.

## 5. Conclusions

This study reviewed the clinical data of 36 patients with culture-proven *Pseudomonas aeruginosa* endophthalmitis. Ocular trauma accounted for 41.7% of *Pseudomonas aeruginosa* endophthalmitis cases, followed by corneal ulcer (25.0%) and postoperative endophthalmitis (13.9%). The outcomes of treatment for *Pseudomonas aeruginosa* endophthalmitis in the current study were poor and are consistent with previous literature on this subject. Sixteen patients underwent evisceration. Only one patient had a visual acuity of 20/400, and the other patients had a visual acuity of CF or below. The cultured *Pseudomonas aeruginosa* was 100% sensitive to gentamicin, tobramycin, amikacin, ciprofloxacin, and levofloxacin. Unfortunately, the sensitivities of *Pseudomonas aeruginosa* to ceftazidime, cefepime, and imipenem decreased.

## Figures and Tables

**Table 1:** Demographic characteristics and outcomes of the 36 patients with *Pseudomonas aeruginosa* endophthalmitis.

No/age/sex	Eye	Clinical setting	Initial VA	Final VA	Concurrent Corneal ulcer	Specimens	Surgery	Intravitreal filling
1/62/F	OS	No	NLP	NA	No	Eye content	Evisceration	NA
2/70/M	OS	Ulceration	LP	LP	Yes	Corneal	No	—
3/54/M	OS	Trauma	HM	HM	No	Vitreous	Intravitreal antibiotics/PPV	BSS
4/48/M	OD	Trauma	HM	20/400	Yes	Vitreous	Intravitreal antibiotics	—
5/30/M	OS	Trauma	HM	HM	No	Corneal	PPV	C3F8
6/19/M	OS	Trauma	LP	LP	No	Vitreous	Intravitreal antibiotics/PPV	Silicone oil
7/40/M	OD	Corneal transplantation	LP	CF	Yes	Corneal	Intravitreal antibiotics/PPV + corneal transplantation	BSS
8/85/M	OD	Phacoemulsification	NLP	NA	No	Vitreous	Intravitreal antibiotics/Evisceration	NA
9/20/M	OS	Trauma	LP	LP	No	Vitreous	PPV	Silicone oil
10/71/M	OS	Ulceration	NLP	NA	Yes	Corneal	Evisceration	NA
11/6/M	OS	Endogenous	LP	CF	No	Vitreous	Intravitreal antibiotics/PPV	Silicone oil
12/46/M	OD	Trauma	NLP	NLP	Yes	Corneal	No	—
13/53/M	OS	Ulceration	LP	NA	Yes	Corneal	Evisceration	NA
14/82/M	OD	Phacoemulsification	LP	LP	No	Vitreous	PPV	Silicone oil
15/52/M	OS	Ulceration	NLP	NA	Yes	Eye content	Evisceration	NA
16/47/M	OS	Trauma	HM	LP	No	Vitreous	Intravitreal antibiotics/PPV	Silicone oil
17/71/F	OD	Endogenous	NLP	NA	No	Vitreous	Evisceration	NA
18/8/F	OD	Trauma	HM	HM	No	Vitreous	PPV	Silicone oil
19/59/F	OD	Trauma	HM	HM	No	Vitreous	PPV	Silicone oil
20/9/F	OD	No	LP	CF	No	Vitreous	PPV	Silicone oil
21/43/M	OD	No	NLP	NA	No	Vitreous	Evisceration	NA
22/76/M	OD	Ulceration	NLP	NA	Yes	Vitreous	Evisceration	NA
23/54/M	OS	Trauma	NLP	NA	Yes	Vitreous	Evisceration	NA
24/37/M	OS	Trauma	HM	HM	No	Vitreous	PPV	Silicone oil
25/60/M	OD	No	NLP	NA	No	Conjunctival sac secretion	Evisceration	NA
26/67/F	OS	Ulceration	NLP	NA	Yes	Vitreous	Evisceration	NA
27/81/F	OS	Ulceration	NLP	NLP	Yes	Corneal	No	—
28/73/M	OS	Trauma	HM	NA	Yes	Vitreous	Evisceration	NA
29/49/M	OD	Trauma	LP	HM	No	Vitreous	PPV	Silicone oil
30/31/M	OD	Endogenous	LP	LP	Yes	Corneal	No	—
31/85/F	OD	Trauma	NLP	NA	Yes	Vitreous	Evisceration	NA
32/56/M	OS	Trauma	HM	HM	Yes	Corneal	Intravitreal antibiotics	—
33/45/M	OD	Ulceration	NLP	NA	Yes	Corneal	Evisceration	NA
34/58/M	OD	Corneal transplantation	LP	HM	Yes	Corneal	No	—
35/74/F	OS	Phacoemulsification	LP	NA	No	Vitreous	Evisceration	NA
36/84/F	OS	Ulceration	NLP	NA	Yes	Vitreous	Evisceration	NA

PPV: pars plana vitrectomy. VA: visual acuity; LP: light perception; NLP: no light perception; HM: hand move; CF: counting fingers; NA: no eyeball; UK: unknown.

**Table 2:** Summary of the 36 patients with *Pseudomonas aeruginosa* endophthalmitis.

Variable	N (%)
Sex	
Male	26 (72.2)
Female	10 (27.8)

Age	
0–20	5 (13.9)
21–40	4 (11.1)
41–60	14 (36.9)
61–85	13 (36.1)

Work	
Retiree	15 (41.7)
Worker	8 (22.2)
Peasant	5 (13.9)
Other	5 (13.9)
Student	3 (8.3)

Clinical setting	
Trauma	15 (41.7)
Corneal ulceration	9 (25.0)
Postoperative endophthalmitis	5 (13.9)
Unknown	4 (11.1)
Endogenous	3 (8.3)

System disease	
No	23 (63.9)
Yes	13 (36.1)

Eye	
OS	19 (52.8)
OD	17 (47.2)

Treatment	
Evisceration	16 (44.4)
PPV	13 (36.1)
No surgery	5 (13.9)
Intravitreal antibiotics only	2 (5.6)

Final visual acuity^*∗*^	
CF or better	4 (20.0)
HM	8 (40.0)
LP/NLP	8 (40.0)

^*∗*^Visual acuity of 20 patients. PPV: pars plana vitrectomy. LP: light perception; NLP: no light perception; HM: hand move; CF: counting fingers.

**Table 3:** Antibacterial resistance of cultured *Pseudomonas aeruginosa*.

	Antibiotic resistance^∗^ rate (*n*, %)
Aminoglycosides	
Neomycin	2/13 (15.4)
Gentamicin	0/29 (0.0)
Tobramycin	0/36 (0.0)
Amikacin	0/30 (0.0)

Macrolides	
Azithromycin	6/6 (100.0)

*β* lactams	
Aztreonam	1/21 (4.8)
Piperacillin	7/29 (24.1)
Piperacillin/Tazobactam	7/29 (24.1)
Cefepime	7/29 (24.1)
Ceftazidime	9/35 (25.7)

Carbapenems	
Meropenem	1/29 (3.4)
Imipenem	1/30 (3.3)

Quinolones	
Ofloxacin	2/8 (25.0)
Ciprofloxacin	0/29 (0.0)
Levofloxacin	0/36 (0.0)

^*∗*^The minimum inhibitory concentration method was applied and “intermediate” and being “sensitive” were both considered sensitive.

## Data Availability

The data that support the findings of this study are available from the corresponding author upon reasonable request.
